# Prenatal Attachment and Perinatal Depression: A Systematic Review

**DOI:** 10.3390/ijerph17082644

**Published:** 2020-04-12

**Authors:** Luca Rollè, Maura Giordano, Fabrizio Santoniccolo, Tommaso Trombetta

**Affiliations:** Department of Psychology, University of Turin, 10124 Torino, Italy; l.rolle@unito.it (L.R.); fabrizio.santonic@edu.unito.it (F.S.); tommaso.trombetta@unito.it (T.T.)

**Keywords:** prenatal attachment, perinatal depression, prenatal depression, postpartum depression, systematic review, fathers, mothers, high-risk pregnancies, pregnancy, postpartum

## Abstract

Pregnancy is a period of complex bio-psychological changes, during which the development of an attachment bond to the fetus takes on a central role. Depressive symptoms are common during this period. Both symptoms of depression and low levels of prenatal attachment are related to negative outcomes in caregivers and infants. Following the Preferred Reporting Items for Systematic Review and Meta-Analysis (PRISMA) statement, this systematic review analyzes and systematizes 41 studies concerning the association between prenatal attachment and perinatal depression. The majority of the studies reported a significant association between the two. Specifically, prenatal depressive symptoms were found to be negatively associated with prenatal attachment. Furthermore, lower levels of prenatal attachment were related to higher postnatal depressive symptoms, although fewer studies assessed this association. While these results were found across different populations, conflicting findings emerged, suggesting they should be interpreted with caution, particularly in male samples and in non-normative pregnancies (e.g., high-risk pregnancies, medically assisted pregnancies, and pregnancies with previous perinatal losses). These results are clinically important for the perinatal screening process and for implementing preventive and treatment programs. However, future studies are needed to further confirm and generalize these results.

## 1. Introduction

Pregnancy represents a psychosomatic event that is at the same time enriching, gratifying, and stressful due to physical, physiological, and psychological changes [1,2]. During gestation, caregivers are required to reorganize their internal experience and adjust their new parental responsibilities, roles, and relationships to old ones [3,4].

In particular, during the first trimester of pregnancy, expectant parents must constantly adapt to new mental representations of themselves and of the future baby, which can create a high level of ambivalence and a sense of disorientation [5,6,7]. During this period, the father may evidence a higher level of preoccupation because his experience is less tangible than the mother’s [8]. The mother plays a key role in the assumption of his paternal identity, mediating and supporting the father’s experience [9]. During the second trimester, parents tend to accept this new physical and psychological situation [10]. Additionally, the father begins to become an active participant instead of just a bystander [4,6].

From the 18th to the 25th week of gestation, perceiving fetal movement enables the mother to differentiate between herself and the fetus and to consider him or her as an agent with needs, forms of communication, and intentionality. During this period, prenatal attachment starts to develop [3,10,11]. While the attachment relationship is based on a bidirectional interaction between primary caregivers and the infant that is established in the first years after childbirth with the aim to provide security, protection, and affect regulation to the child [12,13,14], prenatal attachment refers to an abstract and unidirectional bond between parents and the fetus that develops during pregnancy [15,16,17,18]. Antenatal attachment is about the parent’s affects, cognitions and behaviors toward the fetus, such as name attribution, interaction with the fetus, speaking to the fetus, stroking the belly, prenatal fetal care and physical preparation [15,16,17]. For many years, literature mainly focused on maternal prenatal attachment due to the women’s primary role during pregnancy and their tangible, physical and physiological experience [10,19,20,21]. However, in the last few years, some authors have expressed interest toward the paternal experience of pregnancy, in which mothers have a primary role as mediators in the development of the partner’s fantasies and interactions with the fetus [22,23,24]. Several authors provided a detailed definition of prenatal attachment. Cranley was the first to define it as “the extent to which women engage in behaviors that represent an affiliation and interaction with their unborn child” ([15], p. 282). Müller highlighted the role of fantasies, thoughts, and representations [16]. Condon postulated a hierarchical model of prenatal attachment; here, love is at the core experience of attachment. This is followed by five subjective experiences derived from love and connected with parents’ behaviors [17]: the importance of knowing the fetus, staying with him or her, protecting him or her from physical or mental damages, identifying his or her needs, and avoiding separation from the fetus. According to a more recent definition, prenatal attachment represents a bond between a parent and a fetus characterized by the cognitive, emotional, and mental capacities needed to conceptualize another human being [18].

Many authors underline that low maternal prenatal attachment is associated with low prenatal maternal fetus representations and self-care [25,26,27,28,29] and low postnatal attachment until 24 months postpartum [30,31,32,33,34,35,36,37,38,39]. Additionally, poor parental prenatal attachment has been connected to behavioral and conduct problems, lower cognitive development, and negative socioemotional regulations in early childhood [40,41,42].

During the transition to parenthood, which is a time of intense psychological change, women are also at risk for the onset of perinatal depression [43,44]. Compared to men, women are twice as likely develop this symptomatology [45,46]. Studies have shown that the prevalence of depression among men during the perinatal period is approximately 10%, which is higher than rates of men’s depression in other life stages [47,48].

In high-income societies, the prevalence of perinatal depression varies from 5–25% in pregnant women [49,50,51]. This high variation in prevalence rates is likely due to methodological issues in the research process [51,52,53]. Even if studies thoroughly analyze the postnatal period, prenatal depression, especially during the last phase of pregnancy, seems to have a higher prevalence than postnatal depression [54,55]. Indeed, the prevalence of postnatal depression ranges from 13–19% [51,56,57]. As emerged in some studies, in 50% of women prenatal depression does stabilize and continue in the postnatal period [43,46,58]. For this reason, multiple assessments during the three trimesters are necessary to immediately detect, treat, and reduce depressive symptoms [51,58,59]. Medium-term effects such as poor perinatal attachment until 15 months postpartum, reduced medical check-ups, low fetal development, preterm deliveries and low care of the infant can occur due to prenatal depressive symptoms [34,60,61]. A child’s behavioral, cognitive, linguistic, and emotional problems can persist into school age as a long-term effect of a mother’s perinatal depression [62,63,64]. Furthermore, maternal and paternal antenatal moods are associated with anxiety problems, depression disorders, and antisocial behaviors among adolescents [65,66,67].

In light of the abovementioned findings, prenatal attachment and perinatal depression emerged as particularly prominent factors during the transition to parenthood that can impact familial wellbeing [2,68,69,70,71]. Several studies have found a relation between prenatal attachment and perinatal depression [25,72,73,74,75]. However, some other studies did not identify the same results [76,77,78,79]. The results also vary depending on the population being considered [80,81].

In line with these findings, this paper reviews the scientific literature that is focused on the association between prenatal attachment and perinatal depression and systematizes the results that have emerged. Clarifying the relation between these two variables could be clinically important for the screening process during the perinatal period and for implementing preventive and treatment programs that can address both conditions. Furthermore, exploring this association whilst focusing on different populations (i.e., women, men, and parents undergoing non-normative pregnancies) could be useful for providing individualized treatments.

## 2. Material and Methods

### 2.1. Data Source and Search Strategy

The current systematic review followed the Preferred Reporting Items for Systematic Review and Meta-Analysis (PRISMA) statement [82]. Two independent reviewers searched through EBSCO databases (CINAHL Complete, Family Studies Abstracts, Mental Measurements Yearbook, PsycARTICLES, PsycINFO, Social Sciences Abstracts—H.W. Wilson, Sociology Source Ultimate, Violence & Abuse Abstracts), PubMed, Scopus, and Web of Science (All Databases). They analyzed titles, abstracts, and full texts to find eligible studies published from the beginning of the research databases to November 2019. The following keywords were used (“prenatal attachment” OR “maternal fetal attachment” OR “parental fetal attachment” OR “paternal fetal attachment” OR “pre-partum attachment” OR “antenatal attachment” OR “prenatal bonding” OR “maternal fetal bonding” OR “parental fetal bonding” OR “paternal fetal bonding” OR “pre-partum bonding” OR “antenatal bonding”) AND (“depression”).

### 2.2. Inclusion and Exclusion Criteria

The inclusion criteria for this review were: (1) an assessment of the association between prenatal attachment and perinatal depression, (2) original research paper, (3) published in English. Papers that did not meet these inclusion criteria were excluded. Furthermore, reviews, systematic reviews, and meta-analysis were excluded. No time limit for paper searching was imposed; all the articles published from the beginning of the research database up to November 2019 were considered.

### 2.3. Study Selection and Data Extraction

**Figure 1:**
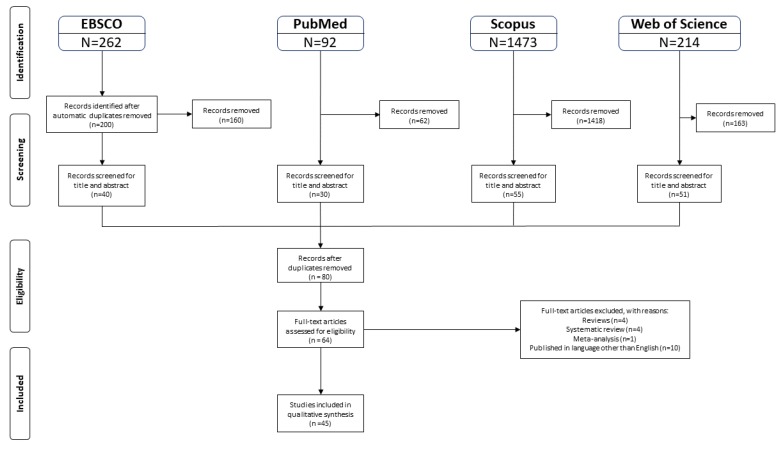
Flowchart of the selection procedure adapted from the Preferred Reporting Items for Systematic reviews and Meta-Analyses (PRISMA) [82].

An initial search of EBSCO yielded 262 results; 40 were selected for full-text review. A second search on PubMed produced 92 results, 30 of which were selected. Scopus yielded 1473 papers, 55 of which were selected. Web of Science provided 214 articles, 51 of which were selected. After removing duplicates, 80 articles in total were obtained. The full texts were analyzed, reducing the number of eligible articles to 64. Of these, 45 studies matched the inclusion and exclusion criteria and were thus included in the current systematic review (Figure 1). Any disagreements between the two independent reviewers (LR and MG) during the study selection and data extraction processes were discussed with the third reviewer (TT), and a unanimous agreement was reached.

### 2.4. Quality Assessment

The quality assessment of the included papers has been conducted by two independent coders using a 15-items quality rating list [83]. Each item can be scored as 1 (yes) or 0 (no/unclear). Any disagreements were discussed to reach a unanimous consensus. The final score of each article has been reported in the last column of Table 1.

## 3. Results

**Figure 2:**
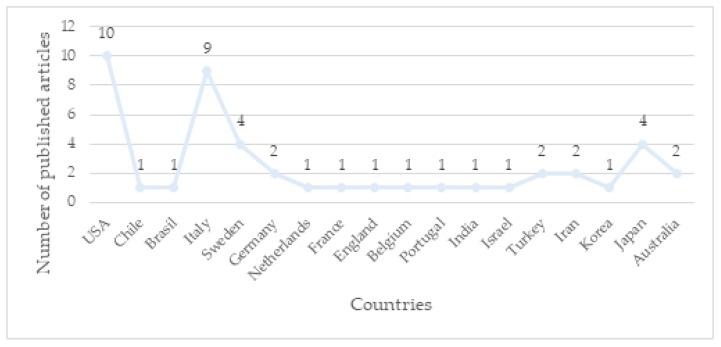
Graph of the number of publications in different countries.

**Figure 3:**
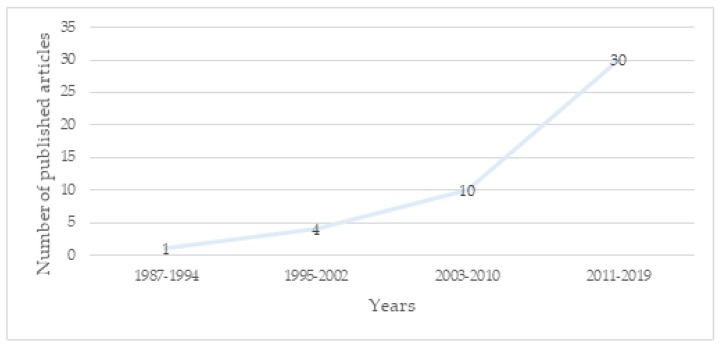
Graph of the number of publications across time.

The included studies were mainly conducted in the US (10 papers) [80,84,85,86,87,88,89,90,91,92] and in Europe (nine papers in Italy [93,94,95,96,97,98,99,100,101], four in Sweden [76,102,103,104], two in Germany [105,106], one in the Netherlands [22], one in France [107], one in England [79], one in Belgium [108], and one in Portugal [75]). Four studies were carried out in Japan [81,109,110,111], two in Australia [112,113], two in Iran [74,114], two in Turkey [78,115], one in India [116], one in Israel [117], one in Chile [118], one in Brazil [119], and one in Korea [120] (Figure 2). All the articles were published between 1988 and 2019 (Figure 3). From 2011 onward, authors’ interest in the relationship between prenatal attachment and depression both during pregnancy and during the first postnatal period increased [74,75,93,94,100,118]. Interestingly, from 2009 onward, an increased number of studies focused on the relationship between prenatal attachment and perinatal depression in fathers in order to facilitate individualized treatments [22,79,103]. Irrespective of the considered time frame, conflicting results emerged regarding the association between the two variables examined in the current systematic review. Nonetheless, it is noteworthy that most of the studies included that failed to find significant results were conducted between 1988 and 2009. During this period, the majority of the studies that explored the association between prenatal attachment and perinatal depression did not find significant results. Instead, between 2010 and 2019 almost three-quarters of the analyzed studies identified results that supported a relationship between the two variables.

### 3.1. Main Findings

Considering the 45 studies included in the current review, 31 found a negative association between prenatal attachment and perinatal depression [22,74,75,81,84,86,89,90,91,92,95,97,98,99,101,102,104,105,106,107,108,110,111,112,114,115,116,117,118,119,120], while a positive relation between perinatal depression and some dimensions of antenatal attachment was identified in three studies [94,102,106]. Thirteen studies did not highlight significant associations between these variables [76,78,79,80,85,87,88,93,96,100,103,109,113]. Specifically, the majority of the articles (28) observed a negative association between prenatal depressive symptoms and prenatal attachment [22,75,81,84,86,89,90,91,92,94,97,98,102,104,105,106,107,108,110,111,112,114,115,116,117,118,119,120]. In contrast, a positive association between prenatal depression and some dimensions of prenatal attachment (i.e., the “physical contact with the fetus and sensitivity to the fetal movement” subscale of the MFAS [102], the “empathy” subscale of the German version of the MFAS [106], and the “fantasy” and the “maternal sensitivity to the fetus” factors of the PAI [94]) was found in three studies [94,102,106]. Thirteen studies [76,78,79,80,85,87,93,96,100,103,109,113] did not observe significant results. Ten papers reported that lower levels of prenatal attachment were associated with higher postnatal depression scores [74,81,95,99,101,105,110,111,116,117]; however, one study [85] did not support the association between these two variables. Four studies analyzed the influence between partner’s depressive symptoms and own prenatal attachment [75,87,97,102]. While two studies did not find significant associations [87,102], two authors identified a negative association between maternal prenatal depressive symptoms and paternal prenatal attachment in uncomplicated [75] and high-risk pregnancies [97].

Nineteen of the 29 studies that administered prenatal attachment instruments in the second or the third trimester of gestation underlined a significant association between the two considered variables [22,74,84,86,90,91,92,95,97,98,101,102,104,105,106,112,115,116,117]: 16 studies confirmed a negative association between prenatal attachment and prenatal depression [22,84,86,90,91,92,97,98,102,104,105,106,112,115,116,117] and six studies identified a negative association between prenatal attachment and postnatal depression [74,95,101,105,116,117], while a positive relation was found between prenatal depression and some factors of prenatal attachment in two studies [102,106]. Even if they assessed prenatal attachment in the recommended gestational period, ten articles did not demonstrate a significant association between prenatal attachment and prenatal depression [76,78,80,85,87,93,96,100,103,113] and one study did not support a relation between prenatal attachment and postnatal depression [85].

Six of the ten studies that assessed prenatal attachment between the first and the second or the third trimester of pregnancy found a significant association with perinatal depression: six studies identified a negative association between prenatal attachment and prenatal depression [81,89,108,110,111,118] and three underlined a significant association between prenatal attachment and postnatal depression [81,110,111]. In contrast, the study of Barone, et al. [94] observed a positive association between prenatal depression and the “fantasy” and “maternal sensitivity to the fetus” factors of the PAI. The remaining three articles did not demonstrate an association between prenatal attachment and prenatal depression [79,88,109]. The only one study that administered prenatal attachment tool in the first trimester of pregnancy confirmed the association between prenatal attachment and postpartum depression [99]. All five studies that did not specify the time at which they evaluated prenatal attachment identified a significant association with prenatal depressive symptoms [75,107,114,119,120].

### 3.2. Sociodemographic Variables

Of the 41 articles that focused on pregnant women [74,75,76,78,80,81,84,85,86,87,88,89,90,91,92,93,94,95,96,97,98,99,100,101,102,104,105,106,107,108,109,110,111,112,113,115,116,117,118,119], 30 studies confirmed a significant association between the two variables [74,75,81,84,86,89,90,91,92,94,95,97,98,99,101,102,104,105,106,107,108,110,111,112,114,115,116,117,118,119]: 23 studies identified a negative relation between antenatal attachment and prenatal depression [75,81,84,86,89,90,91,92,97,98,104,105,107,108,110,111,112,114,115,116,117,118,119], while 10 articles found a negative association between antenatal attachment and postnatal attachment [74,81,95,99,101,105,110,111,116,117]. Three articles observed a positive association between prenatal depression and some dimensions of prenatal attachment [94,102,106]. These latter findings emerged within homogeneous samples of women aged 18 or older. Considering this population, 19 studies found a significant negative association between prenatal attachment and prenatal depression [75,81,84,89,90,92,98,102,104,105,106,107,108,110,111,114,115,116,117], while nine articles identified a negative association between prenatal attachment and postpartum depression [81,95,99,101,105,110,111,116,117]. However, considering similar groups of participants, ten studies did not support the association between prenatal attachment and prenatal depression [76,80,87,88,93,96,97,100,109,113], and one study did not confirm the relation between prenatal attachment and postpartum depression [85]. Prenatal attachment was negatively associated with perinatal depression in four of the five studies that considered women aged 14 or older: three articles found an association between prenatal attachment and prenatal depression [91,118,119] and one article found an association between prenatal attachment and postpartum depression [74]. The association between prenatal attachment and prenatal depression was not confirmed in the study by Ulu, et al. [78].

Regarding socioeconomic characteristics, as stated before the most of the studies reported that the majority of their female participants were highly educated or had a college degree, had full-time employment, had a medium economic income, were involved in a stable relationship, and did not belong to an ethnic minority. Within this population, 22 articles reported a significant association between prenatal attachment and perinatal depression: 17 articles found a negative relation between prenatal attachment and prenatal depression [75,81,84,86,98,102,104,105,106,108,110,111,112,114,115,117,118] and nine articles identified a negative association between prenatal attachment and postnatal depression [74,81,95,99,101,105,110,111,117]. In contrast, three studies observed a positive relation between antenatal depression and some dimension of prenatal attachment [94,102,106]. Ten studies did not find significant results: nine studies regarding the association between prenatal attachment and prenatal depression [76,78,87,88,93,96,97,109,113] and one study regarding the association between prenatal attachment and postpartum depression [85]. Papers that included unemployed women [91,92,107,119], women with a low educational status [116], women with a low socioeconomic status [89,90,91,92], or women belonging to an ethnic minority [90,91,92] found a negative association between prenatal attachment and prenatal depression. One study that investigated African American pregnant women did not confirm the association between prenatal attachment and prenatal depression [80]. One study focused on women with a low educational status confirmed the association between prenatal attachment and postpartum depression [116].

Considering the ten studies [22,75,79,84,87,97,100,102,103,120] that investigated the relation between prenatal attachment and prenatal depression among expectant fathers, a negative association was confirmed in four [22,75,97,120]. All these ten studies [22,75,79,84,87,97,100,102,103,120] were focused on males with a university education who were employed, and above 18 years old. None of the studies explored the association between prenatal attachment and postnatal depression within male samples.

### 3.3. Non-Normative Pregnancies

Regarding the three studies focused on the association between prenatal attachment and prenatal depression in high-risk pregnancies [78,84,97], conflicting results emerged. Two articles did not confirm a relation between the two variables [78,84]. In contrast, Pisoni, et al. [97] observed a negative association between prenatal attachment in men and maternal prenatal depression.

Considering the studies focused on participants undergoing Assisted Reproductive Technology (ART), two research did not observe an association between the two considered variables in either men or women at the 26th and the 36th week of gestation [76,103], while one study identified an association between prenatal attachment and both antenatal and postnatal depression [116].

Within the two studies that considered expectant parents who had dealt with one or more perinatal losses [87,107] (termination of pregnancy, miscarriage, therapeutic abortion, in utero death, or early neonatal death), Gaudet [107] found that higher levels of maternal prenatal depression were related to lower maternal attachment quality. Armstrong [87] found that a partner’s depression was not related to own prenatal attachment.

### 3.4. Psychological Measures

Forty articles were focused on the association between prenatal attachment and prenatal depression, whereas eleven studies analyzed the association between prenatal attachment and postnatal depression [74,81,85,95,99,101,105,110,111,116,117]. All the studies included in the current systematic review (Table 1) [22,74,75,76,78,79,80,81,84,85,86,87,88,89,90,91,92,93,94,95,96,97,98,99,100,101,102,103,104,105,106,107,108,109,110,111,112,113,114,115,116,117,118,119,120] adopted a quantitative approach based on the administration of self-report instruments. One article [91] also used qualitative in-depth interviews with a subsample of 12 women who obtained higher or lower Edinburgh Postnatal Depression Scale (EPDS) scores compared to the cutoff value. These narrative interviews were used to raise the informational representativeness of the quantitative results.

Differences emerged regarding the assessment tools the studies employed. To evaluate maternal prenatal attachment, the most commonly used instrument was the Maternal Antenatal Attachment Scale (MAAS; [16]) (13 studies) [75,80,89,92,95,97,99,100,105,107,108,112,113]. Twelve studies employed the Maternal Fetal Attachment Scale (MFAS; [15]) [74,84,86,88,90,91,96,102,106,114,116,119]. Nine studies used the Prenatal Attachment Inventory (PAI; [17]) [76,78,87,93,94,98,101,104,115], and one study [104] administered its revised version, the PAI-R [121]. Three studies employed the Mother Infant Bonding Questionnaire (MIBQ; [110]) [81,110,111], two used the Antenatal Emotional Attachment Questionnaire (AEAQ; [16]) [117,118], and one study [85] used the Prenatal Maternal Attachment Scale [122]. Finally, one study adopted the Antenatal Maternal Attachment Scale (AMAS; [109]), which was developed to measure antenatal attachment before the second trimester.

Regarding the assessment of paternal prenatal attachment, the following tools were employed: five studies used the Paternal Antenatal Attachment Scale (PAAS; [16] [22,75,79,97,100], three studies used the Paternal Fetal Attachment Scale (PFAS; [15]) [84,102,103], one study used the Paternal Antenatal Inventory (PAI-F; [17]) [87], and one study used the Korean Paternal Fetal Attachment Scale (K-PAFAS; [120]). All the scales except for the K-PAFAS [120] were originally designed to measure maternal experience and were adapted to measure paternal experience.

Regarding the tools used to assess maternal prenatal depressive symptoms, 16 studies used the EPDS [74,76,81,88,89,91,92,99,102,105,106,108,110,111,112,113,123]. Researchers often use this instrument because of its clinical utility to discriminate between psychological wellbeing and symptoms of perinatal depression [112]. Eight studies used the Center for Epidemiologic Studies Depression Scale (CES-D; [124]) [84,87,93,94,96,97,98,117]. Four articles used the Hospital Anxiety Depression Scale (HADS; [125]) [75,104,107,112]. Instruments less commonly employed were: the Beck Depression Inventory ([126]; three studies) [114,115,118], the Zung’s Self Rating Depression Scale (ZSDS; [127]; two studies) [109,112], the Depression Anxiety Stress Scales Short-Form Version (DASS-21; [128]; one study) [80], the Profile of Mood States (POMS-D; [129]; one study) [112], the Hamilton Rating Scale for Depression ([130]; one study) [90], the Matthey Generic Mood Question (MGMQ; [131]; one study) [100], the Depressive Experiences Questionnaire ([132]; one study) [117], the Anxiety, Depression and Stress Scale (ADSS; [133]; one study) [116] and the Brief Symptom Inventory (BSI; [134]; one study) [78].

To assess prenatal depression in expectant fathers, five articles administered the EPDS [22,79,100,102,103,123]. Four studies used the CES-D [84,87,97,120,124]. One study administered the HADS-D [75,125].

To examine maternal postnatal depression, ten articles administered the EPDS [74,81,85,99,101,105,106,110,111,119]. Two studies [95,117] used the CES-D [124]. One study [101] administered the Beck Depression Inventory [126] and one study [116] used the Anxiety, Depression and Stress Scale [133]. No studies assessed postnatal depression in fathers.

Prenatal attachment was assessed between the second and the third trimester of pregnancy in 29 studies [22,74,76,78,80,84,85,86,87,90,91,92,93,95,96,97,98,100,101,102,103,104,105,106,112,113,115,116,117], in accordance with prenatal attachment development and its physical manifestations (e.g., perceptions of fetal movement, body shape changes in the mother, and first morphological echography), which the literature highlights often occurs during this period [32,135,136]. Ten studies evaluated prenatal attachment between the first and the second or the third trimester of gestation [79,81,88,89,94,108,109,110,111,118]. One study [99] administered prenatal attachment tools in the first trimester of pregnancy and five studies did not provide clear information on the subject [75,107,114,119,120].

With reference to perinatal depression, there was high variability in measurement administration time, with measurements administered any time between the first trimester of pregnancy and the 18th month postpartum [74,76,78,80,81,85,86,88,89,90,91,92,93,94,95,96,98,99,100,101,104,105,106,107,108,110,111,112,113,114,115,116,117,118].

Considering the participants involved in the studies, the majority of the papers (35 articles) focused on pregnant women [74,76,78,80,81,85,86,88,89,90,91,92,93,94,95,96,98,99,101,104,105,106,107,108,109,110,111,112,113,115,116,117,118,119]. Six studies considered both future parents [75,84,87,97,100,102], whereas four studies included only fathers [22,79,103,120].

Most of the participants were between 18–49 years old (mean age: 30). Conversely, five studies considered women as young as 14 [74,78,91,118,119]. Regarding socioeconomic characteristics, the majority of the participants had a high school education or a college degree, had full-time employment, had a middle economic income, were involved in a stable relationship, and did not belong to an ethnic minority. On the contrary, very few studies focused on unemployed women [107], women with a low socioeconomic status [90,91], women with a low educational status [116], or women who belonged to a minority group [91].

Finally, three studies observed the relationship between the two considered variables in couples who had high-risk pregnancies [78,84,97]; another three did so in women or men undergoing ART [76,103,116]. Only two papers focused on couples [87,107] who were dealing with one or more perinatal losses—termination of pregnancy, miscarriage, therapeutic abortion, in utero death, or early neonatal death.

## 4. Discussion

The current systematic review aimed to review and systematize the international literature focused on the relationship between prenatal attachment and perinatal depression, with the objective to clarify and discuss the emerging results while providing useful information for clinical purposes. Following inclusion and exclusion criteria, 41 papers were included. A negative association was found in most of the studies included between prenatal depression and prenatal attachment as well as between prenatal attachment and postpartum depression. However, many studies failed to do so, and conflicting results emerged particularly within male samples and expectant parents in high-risk pregnancies.

### 4.1. Association between Prenatal Attachment and Perinatal Depression

Thirty-one of the studies included in this systematic review identified a significant negative association between prenatal attachment and perinatal depression [22,74,75,81,84,86,89,90,91,92,95,97,98,99,101,102,104,105,106,107,108,110,111,112,114,115,116,117,118,119,120]. In contrast, three articles found a positive association between prenatal depression and some dimensions of antenatal attachment (i.e., the “physical contact with the fetus and sensitivity to the fetal movement” subscale of the MFAS [102], the “empathy” subscale of the German version of the MFAS [106], and the “fantasy” and the “maternal sensitivity to the fetus” factors of the PAI [94]. These latter findings can be interpreted in several ways. As stated by some authors [94,102], depressed mothers can be more liable to their body perceptions, feelings, and affects, which should not necessarily be considered positive. As such, higher scores on the sensitivity and fantasy dimensions that emerged among depressed women can be interpreted as negative indicators associated with their distressed condition, rather than the demonstration of a more connection with the fetus. Similarly, greater empathy can be positively related with psychological vulnerability [106]. However, as stated by Seymir, et al. [102], awareness about the fetus and higher perceptions and feelings about him or her, regardless of their quality, can contribute to a greater maternal attention towards the future child that in turn can promote the development of a stronger prenatal bond. Accordingly, these results need to be cautiously considered. Future studies focused on the dimensions of prenatal attachment rather than on its overall score can provide further information and clarify these findings.

Twenty-eight of the 40 studies that assessed the relation between prenatal attachment and prenatal depression identified a negative association between the two variables [22,75,81,84,86,89,90,91,92,94,97,98,102,104,105,106,107,108,110,111,112,114,115,116,117,118,119,120]. These studies generally underlined that high levels of prenatal depression predicted low levels of prenatal attachment [22,75,84,86,89,90,91,92,97,98,102,104,105,106,107,108,112,114,115,116,117,118,119,120], thus focusing on prenatal depression as a predictor of prenatal attachment rather than its consequence. Depressive parents can experience strong feelings of worthlessness and guilt, and less confidence as expectant parents [75,86,90]. This condition can influence parents’ response to pregnancy, particularly their ability for bonding with the fetus [75]. However, as several authors stated, depression inhibited a positive bond from developing but not the attachment’s overall intensity—the parents tended to have negative thoughts and emotions toward the fetus [22,89,112].

Although this association was confirmed in the majority of the included studies focused on the relation between prenatal depression and prenatal attachment, it is necessary to interpret these results with caution due to the many studies that did not confirm these findings. As several authors mentioned, the absence of a significant association could be due to methodological issues—such as assessment time and low sample size—or to confounding variables that were controlled for and that consequently hid the effect of prenatal depression symptoms on prenatal attachment (e.g., social support or personality factors) [79,81,85,88,93,109,111,119].

In addition to the relation often found between prenatal depression and prenatal attachment, many studies (ten out of eleven studies) identified a negative association between prenatal attachment and postnatal depression [74,81,95,99,101,105,110,111,116,117], highlighting the influence of prenatal attachment on parents’ mental wellbeing during the postpartum period. Parents who established a more immediate and internal attachment with the fetus and who attributed emotions or behaviors to him or her were more highly invested in pregnancy, less self-critical and less vulnerable to psychological symptoms [74,105,117].

### 4.2. Administration Time

The majority of the articles (19 out of 29) that assessed the association between prenatal attachment and perinatal depression by administering prenatal attachment measurement tools in the second or the third trimester of pregnancy, in line with the beginning of the perception of fetal movement and the development of prenatal attachment [32,135,136], found a significant negative association between prenatal attachment and perinatal depression: 16 out of 24 studies identified an association between prenatal attachment and prenatal depression [22,84,86,90,91,92,97,98,102,104,105,106,112,115,116,117] and six out of seven studies between prenatal attachment and postnatal depression [74,95,101,105,116,117]. A significant negative association was also found when prenatal attachment tools were administered starting from the first trimester of gestation. Of the studies that evaluated antenatal attachment between the first and the second or third trimester of pregnancy, six out of ten confirmed the relation between prenatal attachment and prenatal depression [81,89,108,110,111,118]; three out of three found an association between prenatal attachment and postnatal depression [81,110,111]. Moreover, the only one study that evaluated prenatal attachment in the first trimester identified a negative relation with postpartum depression [99]. These findings suggest that clinicians can assess both prenatal depression and prenatal attachment also before the second trimester of pregnancy, in order to identify prenatal attachment problems at an early stage, especially in those cases where symptoms of depression emerged. An early detection of antenatal attachment difficulties can also be important to implement interventions aimed at the prevention of depressive symptoms in the postpartum period [81,89,94,99,108,110,111,118]. Finally, all of the studies that assessed prenatal attachment at a not non-specific gestational time point confirmed the association between prenatal attachment and postnatal depression [75,107,114,119,120].

### 4.3. Gender Differences

The studies highlighted a major interest in the association between prenatal attachment and perinatal depression among women [75,84]. The focus on expectant mothers is likely due to their main role during the transition to parenthood. They must combine their new maternal identity and family changes with their previous self-representations and lifestyles [137]. Several authors reported that women interacted more with the fetus as well as faced a greater risk of isolation from their families [25,73,77,138,139]. Previous studies highlighted that some of the most prevalent depressive episodes were reported by women [50,138,140,141]. Women with depressive symptoms tended to report lower caring toward the fetus [119], to become more ambivalent regarding the future baby, to express more negative comments, to dislike their bodies, and to be less involved in pregnancy [6,73,142,143]. It is noteworthy that a significant negative association between prenatal attachment and perinatal depression was found both among women younger than 18 [74,91,118,119] and older than 18 [75,81,84,89,90,92,95,98,99,101,102,104,105,106,107,108,110,111,114,115,116,117].

Despite the main role that women play during pregnancy and in the postpartum period, and despite their risk to incur psychological difficulties, fathers also face psychosocial challenges during these periods. During pregnancy, fathers can become psychologically vulnerable due to the increased amount of time they spend on housework, increased financial and management responsibilities, and lower medical support than their female partners obtain [144,145]. These changes can have a negative impact on paternal mood and on the development and stability of prenatal attachment [22,49,75]. Although fewer of the studies focused on the association between paternal prenatal attachment and paternal prenatal depression [146,147], a significant negative association emerged among fathers in four of the nine studies [22,75,97,120]. No studies explored the relation between paternal prenatal attachment and paternal postnatal depressive symptoms.

To the best of the present researchers’ knowledge, only four studies have focused on the association between a partner’s depressive symptomatology and own prenatal attachment [75,87,97,102]. Although Armstrong [87] and Seimyr, et al. [102] showed the absence of an association between a partner’s depressive symptoms and own prenatal attachment, two studies identified a negative association between maternal prenatal depressive symptoms and paternal prenatal attachment scores [75,97]. These latter findings seem to confirm the key role of the woman for the development of the paternal identity and for the creation of a bond between the expectant father and the fetus [9]. Having a partner with depressive symptoms can imply less support and less involvement during pregnancy, which can decrease the marital adjustment that is required to develop prenatal attachment to a fetus [75,112,148]. These considerations have theoretical and clinical relevance, but they require further confirmation.

### 4.4. Sociodemographic Variables

The majority of the revised studies included women who were married or cohabiting, did not belong to an ethnic minority, who had a middle economic income, and had a high level of education [74,81,84,94,95,99,100,101,104,106,109,110,111,112,114,115,117]. Being in these advantageous conditions was not necessarily a protective factor for their mental health [53,59,78]. Within this population, most of the studies identified a significant association between prenatal attachment and perinatal depression: 17 out of 31 articles found a negative relation between prenatal attachment and prenatal depression (e.g., [75,81,84,86,98,102,104,105,106,108,110,111,112,114,115,117,118]); nine out of ten articles identified a negative association between prenatal attachment and postnatal depression (e.g., [74,81,95,99,101,105,110,111,117]). Women exposed to disadvantaged conditions such as belonging to an ethnic minority (e.g., being African American or Hispanic), with a lower socioeconomic status, or those who were unemployed, or with a low educational status, or who had low incomes were more likely to receive less social support. This can compromise women’s mental health, which in turn can affect antenatal attachment [45,91,116,149,150]. Within this population, seven of the eight studies that assessed the relation between prenatal attachment and prenatal depression identified a negative association between these variables [89,90,91,92,107,116,119].

### 4.5. Non-Normative Pregnancies

It is noteworthy that in women with non-normative pregnancies (high-risk pregnancies or assisted reproduction conceptions), most of the studies did not demonstrate a significant association between prenatal depression and antenatal attachment [76,78,84,97,103]. Only Lamba, et al. [116] observed an association between prenatal attachment and perinatal depression in surrogates [116]. Furthermore, one study did find that maternal prenatal depression negatively influences paternal prenatal attachment in high-risk pregnancies [97]. As Pisoni et al. [97] stated, it can be supposed that lower prenatal attachment scores are more associated with the diagnosis of high-risk pregnancy rather than with perinatal depressive symptoms. Lower levels of antenatal attachment can be considered as the result of a defense mechanism against the probable loss of a fetus. These considerations could provide explanations to the lack of findings emerged within this population.

Considering the two studies [87,107] focused on women who had suffered previous losses, a significant negative association between prenatal depression and prenatal attachment was observed by Gaudet [107]. As stated by several authors, when a mother or a father experience one or more perinatal losses, they bear a heavy burden that can affect their mood and disrupt the creation of an antenatal attachment bond [151,152,153]. However, a partner’s depression was not related to own prenatal attachment in the study by Armstrong [87].

### 4.6. Clinical Implications

The results identified in the current systematic review suggest that clinicians should carry out regular screenings for both depression and attachment quality during pregnancy [72,147,154]. Although conflicting results emerged, the negative association between prenatal attachment and perinatal depression found in most of the studies highlights the need for screening processes and preventive programs at an early stage of pregnancy. Interventions aimed at reducing depressive symptoms that can occur during the gestational period and that can impact the psychological wellbeing of expectant parents and the development of a positive prenatal attachment should be implemented [155,156,157,158,159,160]. To enhance this bond takes on a main role, considering its further influence on postnatal depression (as most of the studies included in the current systematic review reported) [74,81,99,105,110,111], and on other important factors involved in the wellbeing of the both parents and the child [28,29,32,33,34,35,37,38,41,42].

Parental–fetal attachment can be modifiable by specific supporting interventions that are efficacious in promoting the quality of parental bonding [161,162,163,164,165]. These programs for parents-to-be should encourage parents’ fantasies about their baby, their proximity to the future child, and their care of the fetus [102,147].

### 4.7. Strengths and Limitations

This review adds to the current knowledge on the association between prenatal attachment and perinatal depression in men, women, and in high-risk pregnancies. The high number of papers and the focus on different populations clarified the relationship between these variables providing relevant information for clinical use.

However, the findings must be considered in the context of the limitations of this study. First, this systematic review is not a meta-analysis and thus we cannot draw statistical conclusions on its findings. Second, the review only included papers published in English; it excluded results obtained in other languages that could provide a broader comprehension of the relation between prenatal attachment and perinatal depression.

### 4.8. Future Research Directions

First, considering the limited and conflicting data that emerged, further studies would be useful to consider the association between prenatal attachment and perinatal depression in male samples and in non-normative pregnancies more in-depth [22,75,84,97,107,120].

Furthermore, future studies interested in the relation between antenatal attachment and perinatal depression should control for potential confounding variables (e.g., social support or personality factors) that could affect the results, as well as strengthen the methodological design of the research (e.g., involve a higher number of participants) to clarify the conflicting results in some cases emerged in the current systematic review. This would yield clearer conclusions about the association between these variables, which could further orientate healthcare professionals toward implementing rigorous screening processes and interventions during patients’ transition to parenthood.

Moreover, future studies are necessary to better understand the influence of prenatal depressive symptoms on the quality of the partner’s transition to parenthood, which was underexplored in the studies included in the current systematic review [87,102].

In addition, most of the studies included considered subjects with a high socioeconomic status and women who were over 18 years old [75,78,80,81,92,99,101,115]. Only a few studies [90,91,119] analyzed samples containing women as young as 14 years or with socioeconomic disadvantages. Further studies that consider different sociodemographic contexts are necessary to better generalize the present results.

Finally, future systematic reviews should also include results of studies published in language other than English, such as Italian, Japanese, French, and Portuguese, which although they emerged through the databases that we consulted, we did not analyze according to our inclusion and exclusion criteria. This solution can provide a greater understanding of the relationship between prenatal attachment and perinatal depression.

## 5. Conclusions

Analyzing and systematizing the existing literature on the relation between prenatal attachment and perinatal depression, this systematic review found a significant negative association between prenatal depressive symptoms and antenatal attachment and between prenatal attachment quality and postpartum depressive symptoms across different populations in most of the studies it reviewed. However, conflicting results emerged, suggesting that these findings should be interpreted with caution. Further studies are needed to clarify the nature and the generalizability of this association. These results could provide important information for clinical purposes such as implementing screening processes and interventions aimed at reducing the psychological impact of the transition to parenthood and at improving familial wellbeing.

## Figures and Tables

**Table 1:** Studies included in the systematic review.

Authors	Title	Participants	Prenatal Attachment Measurement	Depression Measurement	Conclusions	Quality of the Study (Score out of 15)
Mercer, Ferketich, DeJosepht & Sollid (1988) [84]	Further exploration of maternal and paternal fetal attachment	4 groups: group 1: 153 high-risk women; group 2: 75 male partners mates of high-risk women; group 3: 218 low risk women; group 4: 147 male mates of low risk women.	Maternal Fetal Attachment Scale Paternal Fetal Attachment Scale (MFAS)	Center for Epidemiologic Studies Depression Scale (CES-D)	No associations were found between prenatal depression and prenatal attachment in low risk men, and high-risk women and men. Prenatal depression was related to fetal attachment only among low risk women.	10
Fowles (1996) [85]	Relationships among prenatal maternal attachment, presence of postnatal depressive symptoms, and maternal role attainment	136 pregnant women	Prenatal Maternal Attachment Scale	Edinburgh Postnatal Depression Scale (EPDS)	No significant associations were found between maternal prenatal attachment and postnatal depression.	11
Condon & Corkindale (1997) [112]	The correlates of antenatal attachment in pregnant women	238 pregnant women	Maternal Antenatal Attachment Scale (MAAS)	Edinburgh Postnatal Depression Scale (EPDS) The Hospital Anxiety Depression Scale (HADS-D) The Profile of Mood States (POMS-D) The Zung Self-Rating Depression Scale (ZUNG)-	A negative association was found between prenatal maternal depression and prenatal maternal attachment.	9
Priel & Besser (1999) [117]	Vulnerability to postpartum depressive symptomatology: Dependency, self-criticism and the moderating role of antenatal attachment	73 pregnant women	Antenatal Emotional Attachment Questionnaire (AEAQ)	Center for Epidemiological Studies-Depression Scale (CES-D) Depressive Experiences Questionnaire (DEQ)	A negative association was found between maternal antenatal attachment and prenatal and postnatal depression.	9
Lindgren (2001) [86]	Relationships among maternal-fetal attachment, prenatal depression, and health practices in pregnancy	252 pregnant women	Maternal Fetal Attachment Scale (MFAS)	Center for Epidemiological Studies-Depression Scale (CES-D)	Prenatal depression was a significant predictor of antenatal maternal attachment to the fetus.	13
Honjo, Arai, Kaneko, et al. (2003) [109]	Antenatal Depression and Maternal-Fetal Attachment	216 pregnant women	Antenatal Maternal Attachment Scale (AMAS)	Zung’s Self Rating Depression Scale (ZSDS)	No associations were found between maternal fetal attachment and prenatal depression.	8
Armstrong (2004) [87]	Impact of Prior Perinatal Loss on Subsequent Pregnancies	40 expectant couples with prior perinatal loss	Prenatal Attachment Inventory (PAI-F for fathers)	Center for Epidemiologic Studies Depression Scale (CES-D)	No associations were found between partner’s prenatal depressive symptoms and own prenatal attachment to the fetus in parents with previous perinatal losses.	12
Hart & McMahon (2006) [113]	Mood state and psychological adjustment to pregnancy	53 pregnant women	Maternal Antenatal Attachment Scale (MAAS)	Edinburgh Postnatal Depression Scale (EPDS)	No associations were found between prenatal attachment quality and prenatal depressive symptoms.	9
Hjelmstedt, Widström & Collins (2006) [76]	Psychological Correlates of Prenatal Attachment in Women Who Conceived After In Vitro Fertilization andWomen Who Conceived Naturally	56 IVF women and 41 control women	Prenatal Attachment Inventory (PAI)	Edinburgh Postnatal Depression Scale (EPDS)	No associations were found between prenatal attachment and prenatal depression in IVF women and in control women.	11
Haedt & Keel (2007) [88]	Maternal attachment, depression, and body dissatisfaction in pregnant women	196 pregnant women	Maternal Fetal Attachment Scale (MFAS)	Edinburgh Postnatal Depression Scale (EPDS)	No associations were found between antenatal maternal attachment and prenatal depression.	10
Hjelmstedt, Widström, & Collins (2007) [103]	Prenatal attachment in Swedish IVF fathers and controls	53 of IVF men and 37 of control men at the 26th week of gestation. (Two of the IVF women and two of the control women gave birth before the assessment in the gestational week 36th).	Paternal Fetal Attachment Scale (PFAS)	Edinburgh Postnatal Depression Scale (EPDS)	No associations were found between prenatal paternal attachment and prenatal depression in IVF men and in control men.	9
Brandon, Trivedi, Hynan, et al. (2008) [89]	Prenatal depression in women hospitalized for obstetric risk	129 pregnant women	Maternal Antenatal Attachment Scale (MAAS)	Edinburgh Postnatal Depression Scale (EPDS)	A negative association was found between antenatal attachment and prenatal depression.	11
Seimyr, Sjögren, Welles-Nyström, et al. (2009) [102]	Antenatal maternal depressive mood and parental–fetal attachment at the end of pregnancy	298 pregnant women and 274 partners	Maternal Fetal Attachment Scale (MFAS) Paternal Fetal Attachment Scale (PFAS)	Edinburgh Postnatal Depression Scale (EPDS)	A positive association was found between maternal prenatal depression and physical contact with the fetus and sensitivity to the fetal movements. A negative association was found between perception of woman’s body and pregnancy, and maternal prenatal depression. No associations were found between paternal fetal attachment and maternal prenatal depression.	8
Gaudet (2010) [107]	Pregnancy after perinatal loss: association of grief, anxiety and attachment	96 pregnant women with a prior perinatal loss and 74 pregnant women without a prior perinatal loss	Maternal Antenatal Attachment Scale (MAAS)	The Hospital Anxiety Depression Scale-D (HADS-D)	A negative association was found between prenatal depression and the MAAS quality subscale in women with a previous perinatal loss.	9
van Bussel, Spitz & Demyttenaere (2010) [108]	Reliability and validity of the Dutch version of the maternal antenatal attachment scale	403 pregnant women	Maternal Antenatal Attachment Scale (MAAS)	Edinburgh Postnatal Depression Scale (EPDS)	A negative correlation was found between MAAS quality subscale and EPDS scores at first, second and third trimester of pregnancy. No correlations were found between global MAAS scores or MAAS intensity subscale and depression scores.	12
Della Vedova, Ducceschi, Cesana & Imbasciati (2011) [93]	Maternal bonding and risk of depression in late pregnancy: a survey of Italian nulliparous women	146 pregnant women	Prenatal Attachment Inventory (PAI)	Center for Epidemiological Studies-Depression Scale (CES-D)	No association was found between prenatal attachment and a CES-D score ≥ 16.	13
McFarland, Salisbury, Battle, et al. (2011) [90]	Major depressive disorder during pregnancy and emotional attachment to the fetus	161 pregnant women	Maternal Fetal Attachment Scale (MFAS)	Hamilton Rating Scale for Depression (HRSD)	A negative relationship was found between HRSD scores and MFAS scores.	10
Abasi, Tahmasebi, Zafari, et al. (2012) [114]	Assessment on effective factors of maternal-fetal attachment in pregnant women	386 pregnant women	Maternal Fetal Attachment Scale (MFAS)	Beck Depression Scale (BDI)	Mothers with high prenatal levels of depression had a low antenatal attachment toward the fetus.	9
Alhusen, Gross, Hayat, Rose & Sharps (2012) [91]	The role of mental health on maternal-fetal attachment in low-income women	166 pregnant women (quantitative sample) and a subsample of 12 pregnant women (qualitative sample)	Maternal Fetal Attachment Scale (MFAS) Qualitative Interviews	Edinburgh Postnatal Depression Scale (EPDS) Qualitative Interviews	Prenatal depression was negatively associated to antenatal attachment. In the qualitative subsample, social support mediated the association between the two variables considered.	10
Goecke, Voigt Faschingbauer, et al. (2012) [105]	The association of prenatal attachment and perinatal factors with pre- and postpartum depression in first-time mothers	161 pregnant women	Maternal Antenatal Attachment Scale (MAAS)	Edinburgh Postnatal Depression Scale (EPDS)	Prenatal attachment quality was negatively associated with depression in pregnancy, at the 3rd week postpartum, and at the 6th and 18th month postpartum.	10
Ossa, Bustos & Fernandez (2012) [118]	Prenatal attachment and associated factors during the third trimester of pregnancy in Temuco, Chile	244 pregnant women	Antenatal Emotional Attachment Questionnaire (AEAQ)	Beck Depression Inventory (BDI)	A significant association was found between poor maternal-fetal attachment and high depressive scores.	12
Barone, Lionetti, & Dellagiulia (2014) [94]	Maternal-fetal attachment and its correlates in a sample of Italian women: a study using the Prenatal Attachment Inventory	130 pregnant women	Prenatal Attachment Inventory (PAI)	Center for Epidemiological Studies-Depression Scale (CES-D)	This research highlighted an association between prenatal depressive symptoms and the Fantasy and Sensitivity subscales of the PAI.	9
Diniz, Volling & Koller (2014) [119]	Social support moderates the association between depression and maternal–fetal attachment among pregnant Brazilian adolescents	49 pregnant women	Maternal Fetal Attachment Scale (MFAS)	Edinburgh Postnatal Depression Scale (EPDS)	Prenatal maternal depression scores were associated with prenatal maternal attachment scores only in mothers who reported a high level of social support but not in women with low social support.	11
Vreeswijk, Maas, Rijk & van Bakel (2014) [22]	Fathers’ Experiences During Pregnancy: Paternal Prenatal Attachment and Representations of the Fetus	301 partners of pregnant women	Paternal Antenatal Attachment Scale (PAAS)	Edinburgh Postnatal Depression Scale (EPDS)	A negative correlation was found between depressive symptoms and the quality subscale of the PAAS.	8
Rubertsson, Pallant, Sydsjö, et al. (2015) [104]	Maternal depressive symptoms have a negative impact on prenatal attachment—findings from a Swedish community sample	718 pregnant women	Prenatal Attachment Inventory-R (PAI-R)	Hospital Anxiety and Depression Scale-Depression (HADS-D)	Women with high HADS-D scores (HADS-D > 8) had a high risk of low levels of antenatal attachment in all three PAI-R subscales.	13
Busonera, Cataudella, Lampis, et al. (2016) [95]	Investigating validity and reliability evidence for the maternal antenatal attachment scale in a sample of Italian women	482 pregnant women	Maternal Antenatal Attachment Scale (MAAS)	Center for Epidemiological Studies-Depression Scale (CES-D)	A negative correlation was found between MAAS scores and postnatal depressive symptoms.	10
Busonera, Cataudella, Lampis, et al. (2016) [96]	Psychometric properties of a 20-item version of the Maternal–FetalAttachment Scale in a sample of Italian expectant women	482 pregnant women	Maternal Fetal Attachment Scale (MFAS)	Center for Epidemiological Studies-Depression Scale (CES-D)	No significant correlations were found between prenatal maternal attachment and prenatal depression scores.	10
Pisoni, Garofoli, Tzialla, et al. (2016) [97]	Complexity of parental prenatal attachment during pregnancy at risk for preterm delivery	37 couples without risk of preterm delivery (PP) and 45 couples with risk of preterm delivery (RP)	Maternal Antenatal Attachment Scale (MAAS) Paternal Antenatal Attachment Scale (PAAS)	Center for Epidemiological Studies-Depression Scale (CES-D)	In both groups maternal antenatal attachment did not correlate with prenatal depression. In RP group, but not in PP group, paternal antenatal attachment was negatively correlated with maternal prenatal depression.	8
Busonera, Cataudella, Lampis, et al. (2017) [98]	Prenatal Attachment Inventory: expanding thereliability and validity evidence using a sample ofItalian women	535 pregnant women	Prenatal Attachment Inventory (PAI)	Center for Epidemiological Studies-Depression Scale (CES-D)	PAI scores were negatively correlated with prenatal maternal depressive symptomatology.	11
Noh & Yeom (2017) [120]	Development of the Korean Paternal Fetal Attachment Scale	230 men with pregnant spouse: 30 participants were included in the pilot test and 200 participants in the large survey	Korean Paternal Fetal Attachment Scale(K-PAFAS)	Center for Epidemiological Studies-Depression Scale (CESD-D)	Higher K-PAFAS scores were inversely correlated with CES-D scores in expectant fathers.	9
Ohara, Okada, Kubota, et al. (2017) [110]	Relationship between maternal depression and bondingfailure: A prospective cohort study of pregnant women	751 pregnant women	Mother Infant Bonding Questionnaire (MIBQ)	Edinburgh Postnatal Depression Scale (EPDS)	Prenatal depression, at T_1_ and T_2_, was negatively correlated with MIBQ scores. It was also found a negative correlation between postnatal depression, at T_3_, and MIBQ scores at T_1_ and at T_2_.	9
Ohara, Okada, Aleksic, et al. (2017) [111]	Social support helps protect against perinatal bonding failure and depression among mothers: A prospective cohort study	494 pregnant women	Mother Infant Bonding Questionnaire (MIBQ)	Edinburgh Postnatal Depression Scale (EPDS)	The MIBQ subscales at T_1_ were correlated with postpartum depression at T_2_.	10
Delavari, Mohammad-Alizadeh-Charandabi & Mirghafourvand (2018) [74]	The Relationship of Maternal-Fetal Attachment and Postpartum Depression: A Longitudinal Study	242 pregnant women	Maternal Fetal Attachment Scale (MFAS)	Edinburgh Postnatal Depression Scale (EPDS)	A negative correlation was found between PPD and all MFA’s domains (with exception for the “giving of self” domain)	12
Doster, Wallwiener, Müller, et al. (2018) [106]	Reliability and validity of the German version of the Maternal-Fetal Attachment Scale	324 pregnant women	Maternal Fetal Attachment Scale (MFAS)	Edinburgh Postnatal Depression Scale (EPDS)	Maternal antenatal attachment was not associated with EPDS scores. It was found a positive association between Empathy subscale and depressive symptoms at T_1_ and a negative association between Caring subscale and depressive symptoms at T_1_.	12
Hopkins, Miller, Butler, et al. (2018) [80]	The relation between social support, anxiety and distress symptoms and maternal fetal attachment	94 pregnant women	Maternal Antenatal Attachment Scale (MAAS)	Depression Anxiety Stress Scale-21 (DASS–21)	Prenatal depressive distress was not associated with antenatal attachment.	12
Lamba, Jadva, Kadam et al. (2018) [116]	The psychological well-being and prenatal bonding of gestational surrogates	45 surrogates; 69 pregnant women	Maternal Fetal Attachment Scale (MFAS)	Anxiety, Depression and Stress Scale (ADSS)	A negative association was found between prenatal attachment and prenatal and postnatal depression in surrogates.	12
Petri, Palagini, Bacci, et al. (2018) [99]	Maternal-foetal attachment independently predicts the quality of maternal-infant bonding and post-partum psychopathology	106 pregnant women	Maternal Antenatal Attachment Scale (MAAS)	Edinburgh Postnatal Depression Scale (EPDS) Perinatal Depression Predictor Inventory Revised (PDPI–R)	Maternal-fetal bonding at the 6th months of pregnancy predicted postpartum depressive symptoms.	11
Ulu & Bayraktar (2018) [78]	Investigation of variables related to prenatal bonding levels in pregnant women	200 pregnant women—There are four groups: normal pregnancies in the second trimester, normal pregnancies in the third trimester, risk pregnancies in the second trimester, and risk pregnancies in the third trimester (normal pregnancies: n = 100, % = 50.0 and risk pregnancies: n = 100, % = 50.0)	Prenatal Attachment Inventory (PAI)	Brief Symptom Inventory (BSI)	No associations were found between prenatal maternal bonding and depression.	7
Beesley, Karwatzki & Sullivan (2019) [79]	Anxiety and Depression Symptoms in Fathers During their Partner’s Pregnancy: How does this Impact Paternal Fetal Attachment?	166 fathers	Paternal Antenatal Attachment Scale (PAAS)	Edinburgh Postnatal Depression Scale (EPDS)	This research did not find a significant correlation between prenatal depression and paternal antenatal attachment scores.	13
Brandão, Brites, Pires, & Nunes (2019) [75]	Anxiety, depression, dyadic adjustment, and attachment to the fetus in pregnancy: Actor-partner interdependence mediation analysis	320 pregnant couples	Maternal Antenatal Attachment Scale (MAAS) Paternal Antenatal Attachment Scale (PAAS)	Hospital Anxiety and Depression Scale-Depression (HADS-D)	Depressive symptoms were correlated with maternal antenatal attachment and paternal antenatal attachment. Maternal depressive symptoms in pregnancy were negatively associated with paternal antenatal attachment. Paternal prenatal depressive symptoms were not associated with mother’s antenatal attachment.	11
Della Vedova, Cristini, Bizzi (2019) [100]	Prenatal attachment, distress symptoms and psychosocial variables in a sample of Italian first-time parents	93 couples	Maternal Antenatal Attachment Scale (MAAS); Paternal AntenatalAttachment Scale (PAAS)	Edinburgh Postnatal Depression Scale (EPDS) Matthey Generic Mood Question (MGMQ)	No associations were found between maternal prenatal attachment and prenatal depression.	10
Nagle-Yang, Phillips, Albaugh, et al. (2019) [92]	Depression, anxiety, and attachment among women hospitalized on an antepartum unit	98 pregnant women	Maternal Antenatal Attachment Scale (MAAS	Edinburgh Postnatal Depression Scale (EPDS)	A negative correlation was found between prenatal depression and prenatal attachment scores.	11
Ozcan, Ustundag, Yilmaz et al. (2019) [115]	The Relationships between Prenatal Attachment, Basic Personality Traits, Styles of Coping with Stress, Depression, and Anxiety, and Marital Adjustment Among Women in the Third Trimester of Pregnancy	80 pregnant women	Prenatal Attachment Inven- tory (PAI)	Beck Depression Inventory (BDI)	A negative association was found between maternal prenatal depression and maternal prenatal attachment.	9
Smorti, Ponti, Pancetti (2019) [101]	A Comprehensive Analysis of Post-partum Depression Risk Factors: The Role of Socio-Demographic, Individual, Relational, and DeliveryCharacteristics	161 pregnant women	Prenatal Attachment Inventory (PAI)	Beck Depression Inventory (BDI) Edinburgh Postnatal Depression Scale (EPDS)	A negative association was found between maternal prenatal attachment and postnatal depression.	13
